# Ganglioneuroblastoma associated with neurofibromatosis type 1: a case report with a systematic review

**DOI:** 10.3389/fonc.2026.1822794

**Published:** 2026-06-02

**Authors:** Qi Zhang, Song Su, Guifu Geng, Yuexia Bai, Wandong Hu, Wenchao Zhang, Hongwei Zhang, Guangyu Wang

**Affiliations:** 1 Department of Neurology, Jinan Children’s Hospital (Children’s Hospital Affiliated to Shandong University), Jinan, Shandong, China; 2 Epilepsy Center, Jinan Children’s Hospital (Children’s Hospital Affiliated to Shandong University), Jinan, Shandong, China; 3 Department of Pathology, Jinan Children’s Hospital (Children’s Hospital Affiliated to Shandong University), Jinan, Shandong, China; 4 Department of Neurosurgery, Jinan Children’s Hospital (Children’s Hospital Affiliated to Shandong University), Jinan, Shandong, China

**Keywords:** ganglioneuroblastoma, neurofibromatosis type 1, *NF1* gene, pediatric, tumor

## Abstract

**Introduction:**

Neurofibromatosis type 1 (NF1) is a hereditary disorder characterized by variable clinical manifestations and a predisposition to tumor development. The occurrence of ganglioneuroblastoma(GNB) in patients with NF1 is rare.

**Methods:**

Here, we report a pediatric case and present a review of the relevant literature.

**Results:**

A 3-year-old girl presented with progressively enlarging café-au-lait macules since birth and intermittent back and lumbar discomfort. Imaging revealed a mediastinal mass, which was completely resected. Histopathological examination confirmed GNB, and the patient was subsequently referred for adjuvant chemotherapy. Targeted genetic testing identified a heterozygous deletion spanning exons 1–58 of the *NF1* gene. Although several cases of NF1 associated with GNB have been reported, genotypic data remain limited.

**Discussion:**

The variant identified in this case has not been previously described, thereby expanding the phenotypic and genotypic spectrum of NF1-related tumors.

## Introduction

Neurofibromatosis type 1 (NF1) is an autosomal dominant tumor predisposition disorder caused by pathogenic variants in the *NF1* gene, and most commonly manifests in childhood (1). In 2021, the International Consensus Group on Neurofibromatosis Diagnostic Criteria revised the previous diagnostic criteria. The updated criteria are as follows:

A. The diagnostic criteria for NF1 are met in an individual who does not have a parent diagnosed with NF1 if two or more of the following are present:

- Six or more café-au-lait macules over 5 mm in greatest diameter in prepubertal individuals and over 15 mm in greatest diameter in postpubertal individuals.
- Freckling in the axillary or inguinal regional.
- Two or more neurofibromas of any type or one plexiform neurofibroma.
- Optic pathway glioma.
- Two or more iris Lisch nodules identified by slit lamp examination or two or more choroidal abnormalities (CAs)—defined as bright, patchy nodules imaged by optical coherence tomography (OCT)/near-infrared reflectance (NIR) imaging.
- A distinctive osseous lesion such as sphenoid dysplasia,b anterolateral bowing of the tibia, or pseudarthrosis of a long bone.
- A heterozygous pathogenic NF1 variant with a variant allele fraction of 50% in apparently normal tissue such as white blood cells.

B. A child of a parent who meets the diagnostic criteria specified in A merits a diagnosis of NF1 if one or more of the criteria in A are present (2). Patients with NF1 are predisposed to the development of both neural and non-neural benign and malignant tumors. Several studies have demonstrated that the life expectancy of patients with NF1 is reduced by approximately 8–15 years due to malignancy (3, 4), and the proportion of deaths attributable to malignant tumors is significantly higher than that in the general population (5–7). Currently recognized malignancies associated with NF1 include gliomas, rhabdomyosarcoma, gastrointestinal stromal tumors, malignant peripheral nerve sheath tumors, neuroblastoma (NB), and pheochromocytoma. Among these, intracranial gliomas and malignant peripheral nerve sheath tumors—often arising from plexiform neurofibromas—are the most common. In contrast, NB is relatively uncommon in NF1, and ganglioneuroblastoma(GNB) is particularly rare (8).

NB refers to a group of peripheral neuroblastic tumors and represents the most common extracranial solid malignancy in childhood. These tumors arise from primitive sympathetic neural crest cells, with the adrenal glands being the most frequent primary site. The diagnosis of NB follows a standardized approach, including clinical evaluation, imaging localization, pathological confirmation, molecular characterization and risk stratification. This process typically involves a comprehensive assessment comprising medical history, physical examination, imaging studies, laboratory tests, and pathological biopsy. Genetic testing is recommended when feasible. And risk stratification is conducted based on these findings. Pathological biopsy remains the gold standard for diagnosis. The diagnostic criteria for neuroblastoma (NB) include one of the following two conditions:(1)a definitive pathological diagnosis obtained from tumor tissue under light microscopy.(2) the presence of characteristic neuroblasts (small round cells arranged in nests or rosettes with positive staining for anti-GD2 antibodies) in bone marrow aspiration or biopsy, accompanied by elevated serum neuron-specific enolase(NSE) or urinary catecholamine metabolites (9). According to the revised International Neuroblastoma Pathology Committee classification (INPC), (1) Neuroblastoma is morphologically categorized into four subtypes: Neuroblastoma (Schwannian stroma–poor, characterized by neuroblastic features with minimal Schwannian stroma, in which the proportion of stroma-rich components within the tumor does not exceed 50%). (2) Ganglioneuroblastoma, intermixed (Schwannian stroma–rich, with the combined proportion of ganglioneuromatous components and neuroblastomatous foci exceeding 50% of the total tumor volume). (3) Ganglioneuroma (Schwannian stroma–dominant, composed predominantly of ganglioneuromatous stroma, with scattered, variably distributed differentiating neuroblasts and/or clusters of mature ganglion cells as minor components, and may also contain fully mature ganglion cells). (4) Ganglioneuroblastoma, nodular,(One or more neuroblastomatous nodules may be identified, characterized by schwannian stroma–poor neuroblastic components that are sharply demarcated from the stroma-rich ganglioneuroblastoma, intermixed type (GNBi) or ganglioneuroma components. The neuroblastomatous areas are often surrounded by a fibrous pseudocapsule, and the overall schwannian stroma accounts for more than 50% of the total tumor volume) (10). According to the INPC, which incorporates histopathological subtype, patient age, degree of differentiation, and the mitosis–karyorrhexis index (MKI), ganglioneuroblastoma, intermixed type is classified as favorable histology, whereas the nodular type is considered unfavorable histology (11). Although a few cases of NF1 coexisting with GNB have been reported, the associated genotypic features remain rarely described. Here, we report a case of NF1 concomitant with GNB, analyze clinical phenotype and genetic findings, and provide a review of the relevant literature.

## Case presentation

The patient was a 3-year-6-month-old female who presented with multiple café-au-lait spots scattered across the body since birth. Over time, these macules progressively enlarged in size, and freckling developed in the inguinal regions and axillae. She occasionally complained of mild pain in the back and waist. Prior to this presentation, she had not undergone any medical evaluation or received treatment. The birth history was unremarkable, growth and development were within normal limits. No similar clinical features were observed in either parent.

**Figure 1:**
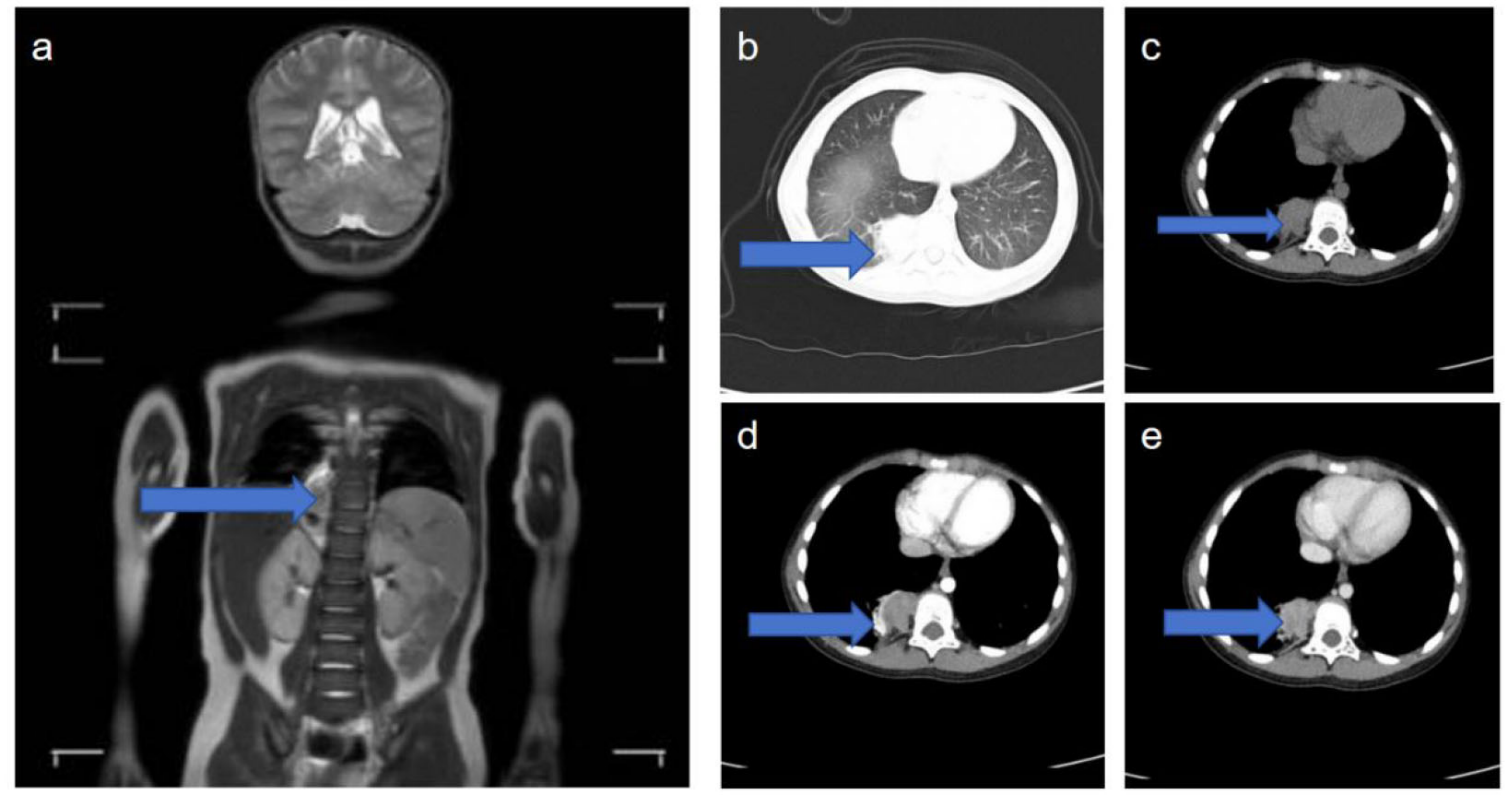
Imaging characteristics of the NF1 patient with GNB. **(a)** Whole - body screening MRI showing the space - occupying lesion in the right mediastinum (arrows). **(b, c)** Non - contrast chest CT images demonstrating the right mediastinal mass (arrows). **(d, e)** Contrast - enhanced chest CT images showing the right mediastinal lesion with heterogeneous enhancement (arrows).

Physical examination, Height: 104 cm; Weight: 16.5 kg. On physical examination, multiple café-au-lait macules were scattered over the body, with more than six lesions measuring >5 mm in diameter, freckling was observed in the inguinal region. No other significant abnormalities were detected on systemic examination. Routine blood tests, cardiac, hepatic, and renal function tests, lipid profile, blood ammonia, lactate, and coagulation function were all within normal limits. Echocardiography and plain radiographs of the long bones showed no abnormalities. Spinal X-ray revealed mild left convex curvature of the thoracic vertebrae, with a Cobb angle of 13°. Ophthalmologic examination identified two Lisch nodules in the left iris. Whole-body screening magnetic resonance imaging (MRI) revealed multiple nodular, slightly hyperintense lesions on T2-weighted images in the bilateral cerebellar hemispheres and bilateral thalamic regions. An ovoid mixed iso-to hyperintense lesion on T2-weighted imaging was observed in the right posterior mediastinum adjacent to the spine, measuring approximately 61.5 × 24.3 mm in the coronal plane. No other significant abnormal signals were detected. The MRI diagnosis indicated intracranial abnormalities consistent with neurofibromatosis type 1 and a space-occupying lesion in the right posterior mediastinum, suggestive of a neurogenic tumor. Contrast-enhanced computed tomography (CT) of the chest and abdomen revealed a soft tissue mass in the right posterior mediastinum, with patchy high-density areas within. Several small nodular shadows were noted around the lesion, which measured approximately 4.3 × 2.5 × 5.1 cm. Contrast enhancement was heterogeneous and pronounced, and intercostal arteries were observed along the margin during the arterial phase. It partially protruded into the thoracic cavity, compressing the adjacent lung tissue, and extended inferiorly behind the diaphragm. No obvious bone abnormalities were observed in the adjacent vertebrae. No significant organic lesions were detected on abdominal pain and enhanced CT (**Figure 1**).

**Figure 2:**
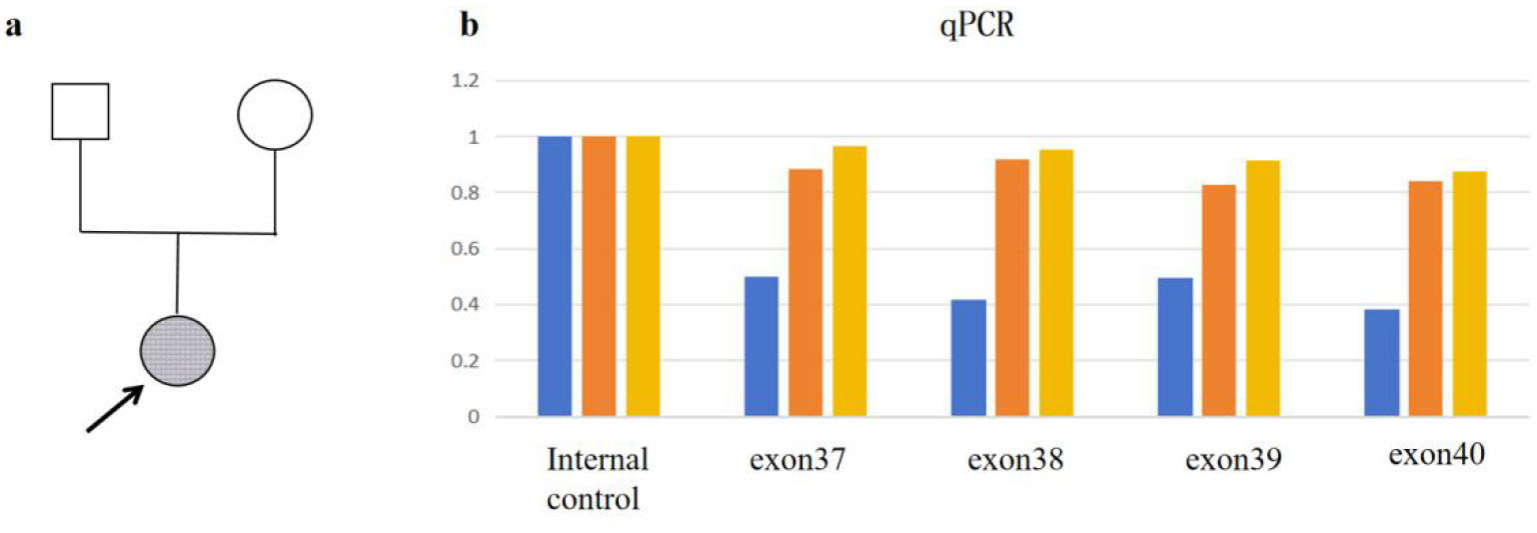
Pedigree and qPCR validation of *NF1* gene deletion. **(a)** Pedigree of the family. The arrow indicates the proband. Both parents are phenotypically unaffected. **(b)** qPCR analysis of four randomly selected exons; the three bar graphs represent the proband, the proband’s mother, and the proband’s father, respectively. A significant copy number variation was detected in the proband, whereas no obvious copy number changes were observed in either parent.

Using high-throughput sequencing, a panel of genes associated with the patient’s clinical phenotype was subjected to deep sequencing and analysis, which revealed that a heterozygous deletion encompassing exons 1–58 of the *NF1* gene was identified. According to the American College of Medical Genetics and Genomics (ACMG) guidelines, the variant was assessed as follows: PVS1, the variant is a null variant (exon deletion), predicted to result in loss of gene function; PS4, the variant has been detected in more than four patients with neurofibromatosis; PM2, the variant is absent from population databases. Based on these criteria, the variant was preliminarily classified as pathogenic. Exons 37–40 were randomly selected for qPCR analysis in the three-member family. In this region, a marked reduction in copy number was detected in the patient, whereas no obvious copy number changes were observed in either parent. The pedigree and qPCR results are shown in the figure (**Figure 2**). The reference genes and primer sequences used for qPCR analysis are provided in **Table 1**.

**Table 1:** Reference genes and primer sequences used for qPCR analysis.

Reference genes and target exons	Forward primer sequence
CRYM	GAAGAACAGACCCGTACCTC
exon 37	GAAATTGTAGTGGACCTTACCC
exon 38	GGTTGGTTTCTGGAGCCTTT
exon 39	AGCTGCCTATAATCTTCTGTGT
exon 40	AATACATGACTCCATGGCTGTC

**Figure 3:**
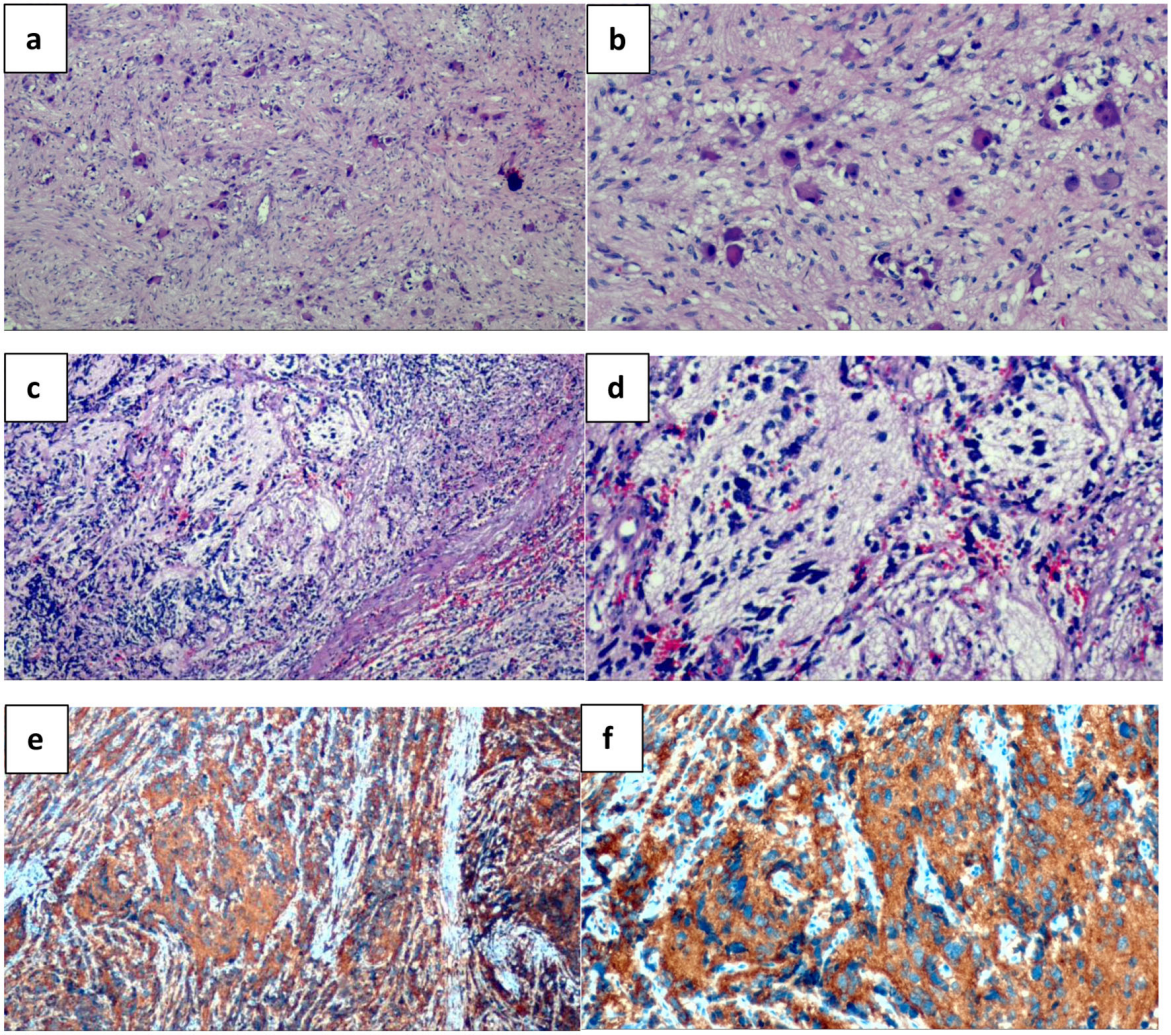
Histopathological and immunohistochemical features of ganglioneuroblastoma, nodular. **(a)** Low-power view showing hypocellular spindle cell proliferation within a collagenous and myxoid stroma, consistent with a neurofibroma-like background (H&E, ×100). **(b)** High-power view demonstrating scattered mature ganglion cells with abundant cytoplasm and prominent nucleoli within the spindle cell stroma (H&E, ×200). **(c)** Nodular architecture with relatively hypercellular areas forming discrete neuroblastic nodules against a less cellular background (H&E, ×100). **(d)** High-power view of the nodular component showing small round blue cells with hyperchromatic nuclei and scant cytoplasm, consistent with neuroblasts (H&E, ×200). **(e)** Immunohistochemical staining for S-100 protein highlighting diffuse positivity in the spindle cell component, supporting Schwannian differentiation (×100). **(f)** Immunohistochemical staining for synaptophysin demonstrating strong cytoplasmic positivity in tumor cells within nodular areas, indicating neuroendocrine differentiation (×200). Overall, the tumor composed of a neurofibroma-like spindle cell stroma and nodular aggregates of neuroblasts with ganglionic differentiation, consistent with nodular ganglioneuroblastoma.

Based on the patient’s clinical manifestations and auxiliary examination results, a preliminary diagnosis was established: (1) Neurofibromatosis type 1; (2) Space-occupying lesion in the right posterior mediastinum. The patient was subsequently transferred to the Department of Thoracic and Oncologic Surgery. After evaluation confirmed no contraindications for surgery, a thoracoscopic mediastinal lesion resection was performed. Intraoperatively, a mass was visualized in the right posterior mediastinum adjacent to the spine, protruding into the thoracic cavity. The mass was dark red, solid, firm in consistency, with poor mobility but relatively clear borders, and it caused compression of the right lung. Upon dissection along the tumor, it was observed to encircle multiple intercostal vessels and nerves. Several enlarged lymph nodes were present around the mass. The tumor, along with the surrounding enlarged lymph nodes, was completely excised by with margins extending beyond the tumor boundary. Postoperative pathology(**Figure 3**) of the right posterior mediastinal mass confirmed ganglioneuroblastoma (nodular type). Metastatic tumor components were identified in the lymph nodes (4/5). Immunohistochemical staining revealed: Syn (+), CgA (+), NSE (+), NF (+), S100 (+), Phox2B (+), PGP9.5 (+), ALK (+), Bcl-2 (+), Ki-67 (40%–50%), CD45 (+), and Phox2B (focal positivity). Following the “Chinese Children’s Cancer Group Neuroblastoma 2021 (CCCG-NB-2021) expert consensus guidelines for the diagnosis and treatment of neuroblastoma” (12), further auxiliary examinations were performed for risk stratification: Fluorescence *in situ* hybridization (FISH) analysis revealed: deletion of 11q23, no deletion of 1p36 gene deletion; MYCN gene gain (The MYCN gene signals were significantly increased relative to the reference signals, with a ratio of less than 4). Neuron - specific enolase (NSE) was mildly elevated at 25.53 μg/L; ferritin, lactate dehydrogenase (LDH), alpha - fetoprotein (AFP), and carcinoembryonic antigen (CEA) were within normal limits. Bone marrow aspiration showed no pathological cells; minimal residual disease (MRD) assessment by flow cytometry revealed no significant immunophenotypically abnormal neuroblastoma cells (residual cells < 5%).

Abdominal, urinary, and retroperitoneal ultrasound revealed no significant abnormalities. Postoperative PET-CT showed increased metabolic activity in the surgical area of the posterior mediastinum, consistent with postoperative changes. Small lymph nodes with increased FDG metabolism in the bilateral cervical, right hilar, and mediastinal regions were considered reactive inflammatory changes. No significant abnormalities in cerebral morphology or FDG metabolism were detected. Karyotype analysis revealed a normal female karyotype (46, XX). Hearing screening was unremarkable. Based on these findings, the final diagnoses were established as: (1) Neurofibromatosis type 1, (2). Ganglioneuroblastoma (nodular type) of the right posterior mediastinum, with 11q23 deletion, is classified as intermediate risk according to the CCCG-NB-2021 protocol.

The patient was subsequently transferred to the Department of Hematology and Oncology to receive standardized chemotherapy according to the CCCG-NB-2021 protocol for intermediate-risk neuroblastoma. As of the last follow-up (January 2026), the patient remains under regular follow-up and is receiving standardized chemotherapy, with no evidence of tumor recurrence.

## Literature review

A systematic search of PubMed, Web of Science, and other databases identified 10 reported cases of NF1 combined with GNB (13–21) (**Table 2**). All 10 patients met the diagnostic criteria for NF1 and had pathologically confirmed GNB. Among them, two cases were classified as the GNB nodular subtype, two as the intermixed subtype, and the remaining six cases did not specify the histological subtype. The adrenal gland was the most common tumor location (6/10 cases). Regarding treatment approaches after a definitive diagnosis: two patients underwent surgical resection of the mass; two patients received chemotherapy alone because of the absence of surgical indications; and five patients received multimodal treatment, including surgery combined with chemoradiotherapy. Post-treatment outcomes showed that five patients achieved complete remission with no evidence of recurrence during regular follow-up of tumor markers and imaging studies; two patients died due to multiple metastases.

**Table 2:** Summary of the present case and previously reported cases of neurofibromatosis type 1 with ganglioneuroblastoma in the literature.

Reference source	Sex	Age at diagnosis	Morphological classification	Tumor location	Treatment	Prognosis
Okonta VN et al	F	5	GNB intermixed	Left adrenal mass	Complete surgical resection of left adrenal mass	No recurrence at 5-year follow-up
Okonta VN et al	M	2	N/A	Adrenal mass	Surgery, radiotherapy, chemotherapy	No recurrence at 1 year post-treatment
Nakagawara A et al	F	14	N/A	Right adrenal (predominantly malignant pheochromocytoma with occasional GNB components, local and regional invasion)	Chemotherapy	Death at 6 months post-treatment due to multiple metastases
Dhermy P et al.	M	4	N/A	Orbital region	Surgery, radiotherapy	N/A
Ito F et al	M	2	N/A	Right renal mesoblastic nephroma, right adrenal GNB	Surgery, chemotherapy	No recurrence at 4 years
Martinsson T et al	N/A	6	N/A	Primary thoracic tumor	Surgery, chemotherapy, radiotherapy, mIBG therapy, retinoic acid	Death at 8 years post-diagnosis due to bone marrow and skeletal system metastases
Dhermy P et al	M	11	GNB intermixed	Right adrenal intramedullary space-occupying lesion	Laparoscopic right adrenalectomy	N/A
Vázquez-Osorio I et al.	F	5	N/A	Abdominal cavity	NA	NA
Puglisi F et al	F	10	GNB nodular	Abdomen	Chemotherapy	Complete remission following chemotherapy
Zhu XJ et al.	M	9	GNB nodular	Left adrenal	Retroperitoneal tumor resection, chemotherapy	No recurrence at 1-year follow-up
Present case	F	3	GNB nodular	right mediastinum	Surgery, chemotherapy	No recurrence was observed during the 6-month follow-up period

Genetic characterization was available for 3 patients. In Case 6, The patient’s father was diagnosed with NF1, whereas the mother exhibited no related clinical features. Genetic analyses identified NF1 variants in both peripheral blood and tumor tissue of the patient. In tumor tissue, a large deletion of the NF1 gene, involving at least exons 39–45, was detected. Subsequent validation by PCR and Southern blot analysis confirmed that the patient’s father harbored the same exon deletion. In contrast, the mother was found to carry a large deletion on chromosome 17 encompassing the region corresponding to the exon loss identified in the patient and her father. This patient presented with a primary thoracic tumor and underwent complete Surgical resection was performed at the initial diagnosis. Over the subsequent eight years, the patient experienced multiple recurrences and received sequential treatments, including surgery, chemotherapy, radiotherapy, mIBG therapy, and retinoic acid. The patient ultimately died from treatment-resistant disease with multiple metastases in the terminal stage. Cases 9 and 10 also reported *NF1* gene variants. Case 10 carried a heterozygous c.1821delG mutation, a frameshift variant, which was also present heterozygously in the patient’s mother.

## Discussion

NF1 is an autosomal dominant disorder characterized by multisystem involvement. Café-au-lait macules are present in approximately 99% of affected individuals, while intertriginous freckling typically develops between 3 and 5 years of age, most commonly in the axillary and inguinal regions. These freckles are smaller than café-au-lait macules, tend to occur in clusters, and have a reported prevalence of approximately 90% (22). In the present study, the patient exhibited multiple café-au-lait macules, intertriginous freckling, and two iris Lisch nodules, all of which fulfill the diagnostic criteria for NF1. NF1 is also a common tumor predisposition syndrome, with affected individuals being susceptible to a wide spectrum of both neural and non-neural benign and malignant neoplasms (23). Malignancy represents one of the leading causes of mortality in these patients (24). Approximately 30%–50% of individuals with NF1 develop plexiform neurofibromas, of which 10%–15% may undergo malignant transformation into malignant peripheral nerve sheath tumors, one of the most frequent malignancies associated with NF1. In addition, other malignancies, including pilocytic astrocytoma, pheochromocytoma, and leukemia, have also been reported (25). In recent years, with the deepening understanding of Neurofibromatosis Type 1, an increasing number of cases complicated by Neuroblastoma have been reported. However, owing to the rarity of this association, most reports remain limited to individual case descriptions. Neuroblastoma is the most common extracranial solid tumor in childhood and arises from primitive neuroectodermal cells. Consequently, it can occur at any site containing embryonic sympathetic ganglion cells and represents a heterogeneous group of diseases with highly variable clinical manifestations and prognosis. In the present study, ganglioneuroblastoma, nodular, represents one of the morphological subtypes in the INPC and is associated with an unfavorable prognosis.

A 3-year-old girl presented with a right posterior mediastinal paravertebral mass identified on imaging. MRI demonstrated a lesion with high signal intensity on T2-weighted images, containing patchy areas of low signal intensity internally. Neurogenic tumors are relatively common in the mediastinum of pediatric patients, and radiologically they should be differentiated from benign peripheral nerve sheath tumors such as plexiform neurofibroma. The latter typically exhibit a distribution along the course of nerves and show a characteristic “target sign” (26) on T2-weighted imaging, defined by a central low signal intensity surrounded by a peripheral high signal intensity. However, in the present case, the internal low-signal areas were irregular and patchy rather than forming a typical target-like configuration, suggesting that the imaging findings were atypical. Further contrast-enhanced CT revealed a localized mass with heterogeneous internal density and evidence of lymph node involvement at the lesion margins. The lesion demonstrated marked heterogeneous enhancement on contrast imaging. These findings are inconsistent with the typically homogeneous low-attenuation appearance of Plexiform neurofibroma (27) and raise suspicion for a malignant neoplasm. Intraoperatively, the tumor appeared dark red and firm, accompanied by enlargement of the surrounding lymph nodes, further suggesting a malignant process. So a definitive diagnosis required further histopathological evaluation.

Histopathological examination revealed a biphasic tumor composition characterized by both immature neuroblastic elements and differentiated components. The immature component consisted of densely packed small round blue cells with hyperchromatic nuclei and scant cytoplasm, forming discrete nodular aggregates. In contrast, the surrounding areas exhibited abundant Schwannian stroma with spindle-shaped cells arranged in fascicular and whorled patterns, admixed with scattered ganglion cells demonstrating abundant eosinophilic cytoplasm and prominent nucleoli. Importantly, the immature neuroblastic nodules were sharply demarcated from the stroma-rich background rather than being diffusely intermixed, supporting a nodular growth pattern. In conjunction with immunohistochemical findings, positivity for Syn, CgA, and NSE supports a neuroendocrine origin of the tumor. Positivity for PGP9.5 and NF further supports an origin from the neural crest or peripheral nerves. NF (neurofilament protein), a cytoskeletal protein predominantly expressed in neurons, indicates neuronal differentiation of the tumor and should not be confused with neurofibromin, the protein product of the *NF1* gene. Positivity for ALK is commonly observed in Neuroblastoma, while PHOX2B is a specific marker of tumors originating from the sympathetic nervous system. Ki-67 is a marker reflecting tumor proliferative activity. In this case, a labeling index of 40–50% indicates a highly proliferative tumor with aggressive biological behavior and a high degree of malignancy. Taken together, the distinct nodular architecture composed of proliferative neuroblastic elements embedded within a Schwannian stroma-rich background, in conjunction with the immunophenotypic profile, supports the diagnosis of ganglioneuroblastoma, nodular.

In addition, the present case should be carefully distinguished from malignant peripheral nerve sheath tumor (MPNST). MPNST (26) predominantly occurs in adults and is frequently associated with NF1. Histologically, it is characterized by fascicles of spindle-shaped cells and typically lacks expression of neuroendocrine markers. In contrast, the tumor cells in this case exhibited a small round cell morphology and showed positive expression for synaptophysin (Syn), chromogranin A (CgA), and PHOX2B, which does not support a diagnosis of MPNST. A systematic search across multiple databases identified a total of 10 reported cases of NF1 coexisting with GNB. All cases met the pathological diagnostic criteria for neuroblastic tumors. However, only 4 had clearly defined histological subtypes, including 2 intermixed and 2 nodular ganglioneuroblastomas. The prognosis of neuroblastic tumors varies widely and is closely associated with age at diagnosis, disease stage, histological subtype, and molecular characteristics (11). Therefore, accurate pathological classification is essential for guiding treatment strategies and determining treatment duration. The present case had a clearly defined histological subtype, thereby contributing to the existing phenotypic spectrum of this rare condition. However, the limited number of reported cases represents a significant limitation. Future studies with larger cohorts are needed to further investigate the relationships between clinical factors—such as sex, age at onset, tumor location, and treatment modalities—and patient outcomes.

NF1 is caused by mutations in the *NF1* gene, which is located on chromosome 17q11.2. The *NF1* gene spans approximately 350 kb and contains 60 exons, including three alternatively spliced exons (28). The gene product, neurofibromin, belongs to the family of mammalian RAS GTPase-activating protein (GAP)-related proteins. Its principal function is to facilitate the conversion of active RAS–GTP to its inactive form, RAS–GDP. Loss of neurofibromin function results in sustained elevation of intracellular RAS–GTP levels, leading to constitutive activation of the RAS–RAF–MAPK signaling pathway and ultimately causing dysregulated cell growth and increased cellular proliferation. In addition, sustained elevation of RAS–GTP levels can activate the PI3K–AKT–mTOR signaling pathway, which promotes cell survival and protects against apoptosis. In the absence of functional neurofibromin, this pathway may remain constitutively activated, leading to enhanced cellular proliferation and contributing to the development of tumor-related features commonly observed in NF1 (29, 30). The mutational spectrum of the NF1 gene reported to date includes missense and nonsense variants, splice-site mutations, microdeletions, microinsertions, insertions/deletions, large deletions, large insertions, and complex rearrangements. Approximately 5%–10% of heritable NF1 variants involve large-scale genomic alterations, most commonly genomic deletions spanning the entire gene and its flanking regions, as well as multi-exon rearrangements (29). In the present study, somatic genetic analysis identified a large deletion encompassing exons 1–58 of the NF1 gene, consistent with the known mutational spectrum. Notably, this variant has not been previously reported, thereby expanding the spectrum of NF1 mutations. Due to the presence of a large exon deletion, qPCR amplification was limited to a subset of regions, and only partial familial validation could be performed because of limited sample availability. Based on the available results, together with the absence of relevant clinical features in the parents, the identified variant in this patient is likely *de novo*. This represents another limitation of the present study. In case 6 identified in our literature review (17), genetic analysis of tumor tissue revealed a large deletion in the NF1 gene involving at least exons 39–45. qPCR and Southern blot analyses confirmed that the patient’s father harbored the same exon deletion, while a large deletion on chromosome 17—encompassing the region corresponding to the exon loss identified in the patient and her father—was detected in the mother. These findings demonstrated a homozygous deletion in the tumor tissue, which was considered to contribute to the development of Ganglioneuroblastoma in this patient. Building on previous findings, and to further evaluate whether this represents a coincidental event in NB, a larger cohort of patients was subsequently analyzed. No similar deletions were identified, and neither familial nor *de novo* cases of NF1 were detected. Although no positive findings were identified, Van Roy N et al. (31) reported that the 17q chromosomal arm in NB is genomically unstable and prone to rearrangements. Bown N et al. (32) further demonstrated that 17q abnormalities, compared with MYCN status, represent a more independent prognostic factor, possibly related to unbalanced translocations involving chromosomes 1 and 17. Accordingly, Martinsson T et al. (17) proposed that the possibility of chromosomal breakpoints occurring near or even within the *NF1* gene on chromosome 17 cannot be excluded. They further suggested that undetected microalterations within the *NF1* gene may exist. Therefore, the contribution of NF1 alterations to the development of GNB in patients with NF1 cannot be definitively ruled out. In the present study, a heterozygous somatic *NF1* gene variant was identified. However, as *NF1* gene analysis was not performed in the tumor tissue, it remains unclear whether a second-hit event occurred, resulting in biallelic inactivation of *NF1* gene within the tumor and complete loss of neurofibromin function, thereby contributing to tumorigenesis. He et al. (33) established a transgenic zebrafish model and demonstrated that *NF1* gene loss cooperates with MYCN oncogene overexpression in Neuroblastoma, promoting tumor cell proliferation and accelerating tumorigenesis. These findings suggest that such a synergistic effect may represent one of the underlying mechanisms contributing to tumor development. Including the present case, only four patients have undergone relevant genetic testing. Whether different types of genetic alterations influence prognosis remains unclear, and no definitive conclusions can be drawn at present. Future studies with larger cohorts are needed to establish databases that enable comprehensive analyses of genotype–prognosis correlations.

Management of patients with NF1 requires a multidisciplinary approach, involving specialties such as dermatology, plastic surgery, oncology, neurosurgery, radiology, ophthalmology, orthopedics, neurology, cardiology, pediatrics, endocrinology, and genetics. Surgical resection remains the primary treatment for tumors that are large or cause functional impairment. For patients with symptomatic, unresectable plexiform neurofibromas, targeted therapy with MEK inhibitors may be considered. Other tumor-related conditions can be managed with surgery, chemotherapy, and/or radiotherapy, depending on the specific tumor type. In the treatment of NB, current clinical practice in China generally follows the CCCG-NB-2021 protocol. In the present study, the patient underwent surgical resection followed by chemotherapy and is currently receiving maintenance chemotherapy. To date, no evidence of recurrence has been observed on regular follow-up, although continued long-term surveillance is warranted. In addition, approximately 80% of relapsed NB cases harbor mutations that lead to activation of the RAS–MAPK signaling pathway. Targeted therapies, including MEK inhibitors and other agents directed against the RAS–MAPK pathway, may suppress the growth of resistant clones. He et al. (33) further conducted pharmacological studies in a zebrafish model and demonstrated that the combination of a MEK inhibitor with isotretinoin significantly inhibited RAS–MAPK pathway activity. As previously described, loss of neurofibromin function due to *NF1* gene mutations in patients with NF1 leads to sustained activation of the RAS–RAF–MAPK signaling pathway. Therefore, MEK inhibitors may represent a potential therapeutic strategy for patients with NF1 complicated by GNB in the future.

## Conclusions

In summary, we report a case of concomitant NF1 and GNB, with the tumor classified as nodular GNB. Somatic genetic analysis revealed a large deletion involving multiple exons of the *NF1* gene. Based on qPCR findings, this variant is presumed to be *de novo*. The identification of this large exon deletion expands both the phenotypic and genotypic spectrum of NF1 associated with GNB. Further studies with larger cohorts are warranted to better elucidate genotype–phenotype correlations in patients with NF1 complicated by GNB.

## Data Availability

The original contributions presented in the study are included in the article/supplementary material. Further inquiries can be directed to the corresponding author.

## References

[1] Wu F Ji XN Chen Q . Pathogenesis and therapeutic advances in neurofibromatosis type 1. Chin J Pediatr. (2023) 61:757–60. doi: 10.3760/cma.j.cn112140-20230227-00136 37528024

[2] Legius E Messiaen L Wolkenstein P Pancza P Avery RA Berman Y . Revised diagnostic criteria for neurofibromatosis type 1 and Legius syndrome: an international consensus recommendation. Genet Med. (2021) 23:1506–13. doi: 10.1038/s41436-021-01170-5 PMC8354850 34012067

[3] Seminog OO Goldacre MJ . Risk of benign tumours of nervous system, and of Malignant neoplasms, in people with neurofibromatosis: population-based record-linkage study. Br J Cancer. (2013) 108:193–8. doi: 10.1038/bjc.2012.535. PMID: 23257896 PMC3553528

[4] Rasmussen SA Yang Q Friedman JM . Mortality in neurofibromatosis 1: an analysis using U.S. death certificates. Am J Hum Genet. (2001) 68:1110–8. doi: 10.1086/320121. PMID: 11283797 PMC1226092

[5] Sorensen SA Mulvihill JJ Nielsen A . Long-term follow-up of von Recklinghausen neurofibromatosis. Survival and Malignant neoplasms. N Engl J Med. (1986) 314:1010–5. doi: 10.1056/NEJM198604173141603 3083258

[6] Duong TA Sbidian E Valeyrie-Allanore L Vialette C Ferkal S Hadj-Rabia S . Mortality associated with neurofibromatosis 1: a cohort study of 1895 patients in 1980–2006 in France. Orphanet J Rare Dis. (2011) 6:18. doi: 10.1186/1750-1172-6-18. PMID: 21542925 PMC3095535

[7] Evans DG O’Hara C Wilding A Ingham SL Howard E Dawson J . Mortality in neurofibromatosis 1: in North West England: an assessment of actuarial survival in a region of the UK since 1989. Eur J Hum Genet. (2011) 19:1187–91. doi: 10.1038/ejhg.2011.113. PMID: 21694737 PMC3198144

[8] Yohay K . Neurofibromatosis type 1 and associated Malignancies. Curr Neurol Neurosci Rep. (2009) 9:247–53. doi: 10.1007/s11910-009-0036-3. PMID: 19348714

[9] Brodeur GM Pritchard J Berthold F Carlsen NL Castel V Castelberry RP . Revisions of the international criteria for neuroblastoma diagnosis, staging, and response to treatment. J Clin Oncol. (1993) 11:1466–77. doi: 10.1200/JCO.1993.11.8.1466. PMID: 8336186

[10] Peuchmaur M d'Amore ES Joshi VV Hata J Roald B Dehner LP . Revision of the International Neuroblastoma Pathology Classification: confirmation of favorable and unfavorable prognostic subsets in ganglioneuroblastoma, nodular. Cancer. (2003) 98:2274–81. doi: 10.1002/cncr.11773. PMID: 14601099

[11] Yang QS Zishi Pediatric Oncology Expert Group . Guidelines for the diagnosis and treatment of pediatric neuroblastoma (2026 edition). J Chongqing Med Univ. (2026) 51:168–77. doi: 10.13406/j.cnki.cyxb.004040

[12] Chinese Anti-Cancer Association Pediatric Oncology Committee Tumor Group, Pediatric Surgery Branch, Chinese Medical Association . Expert consensus on the diagnosis and treatment of pediatric neuroblastoma: CCCG-NB-2021 protocol. Chin J Pediatr Surg. (2022) 43:588–98. doi: 10.3760/cma.j.cn421158-20211227-00638

[13] Okonta VN Majlessipour F Baca NM . Ganglioneuroblastoma in a child with neurofibromatosis type 1: a case report and literature review. J Pediatr Hematol Oncol. (2023) 45:e131–4. doi: 10.1097/mph.0000000000002461. PMID: 35398860

[14] Nakagawara A Ikeda K Tsuneyoshi M Daimaru Y Enjoji M . Malignant pheochromocytoma with ganglioneuroblastoma elements in a patient with von Recklinghausen's disease. Cancer. (1985) 55:2794–8. doi: 10.1002/1097-0142(19850615)55:12<2794::aid-cncr2820551213>3.0.co;2-l 3922614

[15] Dhermy P Sekkat A Moussaoui M Bellakhdar N Haye C Charlot JC . Ganglioneuroblastome de l'orbite [Ganglioneuroblastoma of the orbit. J Fr Ophtalmol. (1985) 8:139–46. 3924990

[16] Ito F Watanabe Y Ito T . Synchronous occurrence of Wilms tumor and ganglioneuroblastoma in a child with neurofibromatosis. Eur J Pediatr Surg. (1997) 7:308–10. doi: 10.1055/s-2008-1071180. PMID: 9402494

[17] Martinsson T Sjöberg RM Hedborg F Kogner P . Homozygous deletion of the neurofibromatosis-1 gene in the tumor of a patient with neuroblastoma. Cancer Genet Cytogenet. (1997) 95:183–9. doi: 10.1016/s0165-4608(96)00259-2. PMID: 9169039

[18] Geraci AP de Csepel J Shlasko E Wallace SA . Ganglioneuroblastoma and ganglioneuroma in association with neurofibromatosis type I: report of three cases. J Child Neurol. (1998) 13:356–8. doi: 10.1177/088307389801300712. PMID: 9701489

[19] Vázquez-Osorio I Duat-Rodríguez A García-Martínez FJ Torrelo A Noguera-Morel L Hernández-Martín A . Cutaneous and systemic findings in mosaic neurofibromatosis type 1. Pediatr Dermatol. (2017) 34:271–6. doi: 10.1111/pde.13094. PMID: 28318056

[20] Puglisi F Soma R Podda M Vetrella S Rabusin M Tropia S . Neuroblastic tumors and neurofibromatosis type 1: a retrospective multicenter study in Italy and systematic review of the literature. Front Pediatr. (2022) 10:950911. doi: 10.3389/fped.2022.950911. PMID: 36405824 PMC9673013

[21] Zhu XJ Xu YR Gu NN . A case of neurofibromatosis type 1 complicated by ganglioneuroblastoma. Chin Med J. (2023) 103:1868–9. doi: 10.3760/cma.j.cn112137-20230110-00054

[22] Hirbe AC Gutmann DH . Neurofibromatosis type 1 a multidisciplinary approach to care. Lancet Neurol. (2014) 13:834–43. doi: 10.1016/s1474-4422(14)70063-8. PMID: 25030515

[23] Cimino PJ Gutmann DH . Neurofibromatosis type 1. Handb Clin Neurol. (2018) 148:799–811. doi: 10.1016/B978-0-444-64076-5.00051-X 29478615

[24] Varan A Şen H Aydın B Yalçın B Kutluk T Akyüz C . Neurofibromatosis type 1 and Malignancy in childhood. Clin Genet. (2016) 89:341–5. doi: 10.1111/cge.12625. PMID: 26073032

[25] Rosenbaum T Wimmer K . Neurofibromatosis type 1 (NF1) and associated tumors. Klin Padiatr. (2014) 226:309–15. doi: 10.1055/s-0034-1382021. PMID: 25062113

[26] Chinese Orthopaedic Association, Neurofibromatosis Group . Expert consensus on the comprehensive management of plexiform neurofibromatosis (2025 edition). Chin Med J. (2025) 105:331–45. doi: 10.3760/cma.j.cn112137-20240809-01824

[27] Bass JC Korobkin M Francis IR Ellis JH Cohan RH . Retroperitoneal plexiform neurofibromas: CT findings. AJR Am J Roentgenol. (1994) 163:617–20. doi: 10.2214/ajr.163.3.8079855. PMID: 8079855

[28] Viskochil D Buchberg AM Xu G Cawthon RM Stevens J Wolff RK . Deletions and a translocation interrupt a cloned gene at the neurofibromatosis type 1 locus. Cell. (1990) 62:187–92. doi: 10.1016/0092-8674(90)90252-a. PMID: 1694727

[29] Philpott C Tovell H Frayling IM Cooper DN Upadhyaya M . The NF1 somatic mutational landscape in sporadic human cancers. Hum Genomics. (2017) 11:13. doi: 10.1186/s40246-017-0109-3. PMID: 28637487 PMC5480124

[30] Brems H Beert E de Ravel T Legius E . Mechanisms in the pathogenesis of Malignant tumours in neurofibromatosis type 1. Lancet Oncol. (2009) 10:508–15. doi: 10.1016/s1470-2045(09)70033-6. PMID: 19410195

[31] Van Roy N Laureys G Cheng NC Willem P Opdenakker G Versteeg R . 1;17 translocations and other chromosome 17 rearrangements in human primary neuroblastoma tumors and cell lines. Genes Chromosomes Cancer. (1994) 10:103–14. doi: 10.1002/gcc.2870100205. PMID: 7520263

[32] Bown N Cotterill S Lastowska M O'Neill S Pearson AD Plantaz D . Gain of chromosome arm 17q and adverse outcome in patients with neuroblastoma. N Engl J Med. (1999) 340:1954–61. doi: 10.1056/nejm199906243402504. PMID: 10379019

[33] He S Mansour MR Zimmerman MW Ki DH Layden HM Akahane K . Synergy between loss of NF1 and overexpression of MYCN in neuroblastoma is mediated by the GAP-related domain. Elife. (2016) 5:e14713. doi: 10.7554/elife.14713. PMID: 27130733 PMC4900799

